# Multi-component prime-boost *Chlamydia trachomatis* vaccination regimes induce antibody and T cell responses and accelerate clearance of infection in a non-human primate model

**DOI:** 10.3389/fimmu.2022.1057375

**Published:** 2022-11-23

**Authors:** Emma Lorenzen, Vanessa Contreras, Anja W. Olsen, Peter Andersen, Delphine Desjardins, Ida Rosenkrands, Helene Bæk Juel, Benoit Delache, Sebastien Langlois, Constance Delaugerre, Christophe Joubert, Nathalie Dereuddre-Bosquet, Cécile Bébéar, Bertille De Barbeyrac, Arabella Touati, Paul F. McKay, Robin J. Shattock, Roger Le Grand, Frank Follmann, Jes Dietrich

**Affiliations:** ^1^ Chlamydia Vaccine Research, Department of Infectious Disease Immunology, Statens Serum Institut, Copenhagen, Denmark; ^2^ Université Paris-Saclay, Inserm, Commissariat à l'Énergie Atomique et aux Énergies Alternatives (CEA), Center for Immunology of Viral, Auto-immune, Hematological and Bacterial diseases (IMVA-HB/IDMIT), Fontenay-aux-Roses & Le Kremlin-Bicêtre, France; ^3^ Novo Nordisk Foundation, Infectious Disease, Hellerup, Denmark; ^4^ Novo Nordisk Foundation, Center for Basic Metabolic Research, Copenhagen, Denmark; ^5^ Laboratory of Virology, Hôpital Saint-Louis, Assistance Publique Hôpitaux de Paris, Université de Paris, Paris Cité, Paris, France; ^6^ Bordeaux University Hopsital, Bacteriology Department, National Reference Centre for bacterial Sexually Transmitted Infections, Bordeaux, France; ^7^ Department of Medicine, Imperial College London, St Mary’s Campus, London, United Kingdom

**Keywords:** vaccine, infection, immunology, bacteria, CD4/CD8 lymphocytes, Chlamydia trachomatis

## Abstract

It is of international priority to develop a vaccine against sexually transmitted *Chlamydia trachomatis* infections to combat the continued global spread of the infection. The optimal immunization strategy still remains to be fully elucidated. The aim of this study was to evaluate immunization strategies in a nonhuman primate (NHP) model. Cynomolgus macaques (*Macaqua fascicularis*) were immunized following different multi-component prime-boost immunization-schedules and subsequently challenged with *C. trachomatis* SvD in the lower genital tract. The immunization antigens included the recombinant protein antigen CTH522 adjuvanted with CAF01 or aluminium hydroxide, MOMP DNA antigen and MOMP vector antigens (HuAd5 MOMP and MVA MOMP). All antigen constructs were highly immunogenic raising significant systemic *C. trachomatis*-specific IgG responses. In particularly the CTH522 protein vaccinated groups raised a fast and strong pecificsIgG in serum. The mapping of specific B cell epitopes within the MOMP showed that all vaccinated groups, recognized epitopes near or within the variable domains (VD) of MOMP, with a consistent VD4 response in all animals. Furthermore, serum from all vaccinated groups were able to *in vitro* neutralize both SvD, SvE and SvF. Antibody responses were reflected on the vaginal and ocular mucosa, which showed detectable levels of IgG. Vaccines also induced *C. trachomatis-*specific cell mediated responses, as shown by *in vitro* stimulation and intracellular cytokine staining of peripheral blood mononuclear cells (PBMCs). In general, the protein (CTH522) vaccinated groups established a multifunctional CD4 T cell response, whereas the DNA and Vector vaccinated groups also established a CD8 T cells response. Following vaginal challenge with *C. trachomatis* SvD, several of the vaccinated groups showed accelerated clearance of the infection, but especially the DNA group, boosted with CAF01 adjuvanted CTH522 to achieve a balanced CD4/CD8 T cell response combined with an IgG response, showed accelerated clearance of the infection.

## Introduction

Genital *Chlamydia trachomatis* infections annually cause an estimated 131 million new cases worldwide (1) and constitutes a major global health issue by potentially inducing severe complications such as pelvic inflammatory disease, ectopic pregnancies and infertility (2, 3). Antibiotics still offer an effective treatment of *C. trachomatis* infections. However, since the infection is asymptomatic in up to 75% of the cases, the majority of infections are left untreated, and transmission is not interrupted (3). It is generally agreed that the most effective weapon against the continued spread of *Chlamydia* infections, is a prophylactic vaccine (4).

Despite several years of research, a vaccine is not yet on the market. This is partly due to the complex bi-phasic lifestyle of *C. trachomatis.* The unique biphasic life cycle of *C. trachomatis*, with both an intracellular and extracellular stage, challenges the immune system. It is believed that protective immunity should consist of cell-mediated immune (CMI) responses, in addition to serotype specific antibodies (5–13). Moreover, generating mucosal immunity in the genital tract poses an additional challenge for a vaccine.

In the present study, we tested different vaccine strategies in an NHP chlamydia infection model. The strategies aimed to induce antibodies, CD4 T cells and/or a CD8 T cell response. In addition, some of the vaccines aimed at generating a mucosal response. The antigens chosen for this study were based on the Major Outer Membrane Protein. Firstly, a consensus MOMP Antigen (Con E) based on approximately 1500 serovar E ompA sequences generated to provide high epitope coverage against the most prevalent *C. trachomatis* strains (14). Secondly, a synthetic fusion protein, CTH522, was used. CTH522 is a recombinant, engineered version of the MOMP, comprising heterologous immunorepeats of VD4 regions from four genital *C.t.* serovars (D, E, F, and G) (11). The strategy behind the recombinant fusion protein is to induce amplified responses to the protective epitopes and induce cross-serovar protection (11).

Another key feature of the current experiment was a parallel assessment of CTH522 in two markedly different adjuvant systems (AlOH and CAF01), combined with an intranasal booster. This combination has been tested in mice and mini-pigs where it was shown to be highly immunogenic by inducing high titers of neutralizing antibodies, IFN-γ producing CD4+ T cells and accelerated clearance of a genital *C. trachomatis* infection (11–13, 15). Furthermore, this comparison mirrored a recent first-in-human clinical trial were both formulations proved to be immunogenic, and where CAF01 was superior in inducing neutralizing serum antibodies and Th1 cell-mediated immune responses (16). To induce strong cellular immune responses, and specifically CD8 T cell responses combined with CD4 T cell responses, part of the animals were boosted with the CT522/CAF01 immunogen after priming with an Adeno vector pAL1112 (HuAd5-MOMP), an MVA pox vector, or a plasmid DNA pcDNA3.1 delivering the MOMP Con E antigen (14, 17). All three vectors have proven immunogenic in NHPs, and it was recently shown that an immunization strategy combining these three vaccines and a final booster vaccination with adjuvanted protein antigen induced superior immune responses and consistently enhanced the clearance of intravaginal *C. trachomatis* in a mouse *C. trachomatis* infection model (14).

In the present study we demonstrate that different combinations of multi-component immunization strategies with both protein vaccines, DNA vaccines and recombinant viral vector vaccines in a nonhuman primate (NHP) model, result in distinct immune responses. Furthermore, we observe that a regimen using DNA priming and protein (CTH522/CAF01) boosting accelerated bacterial clearance following a genital challenge with *Chlamydia trachomatis.*

## Results

### All vaccination regimes proved safe and induced significant systemic antibody responses

**Figure 1:**
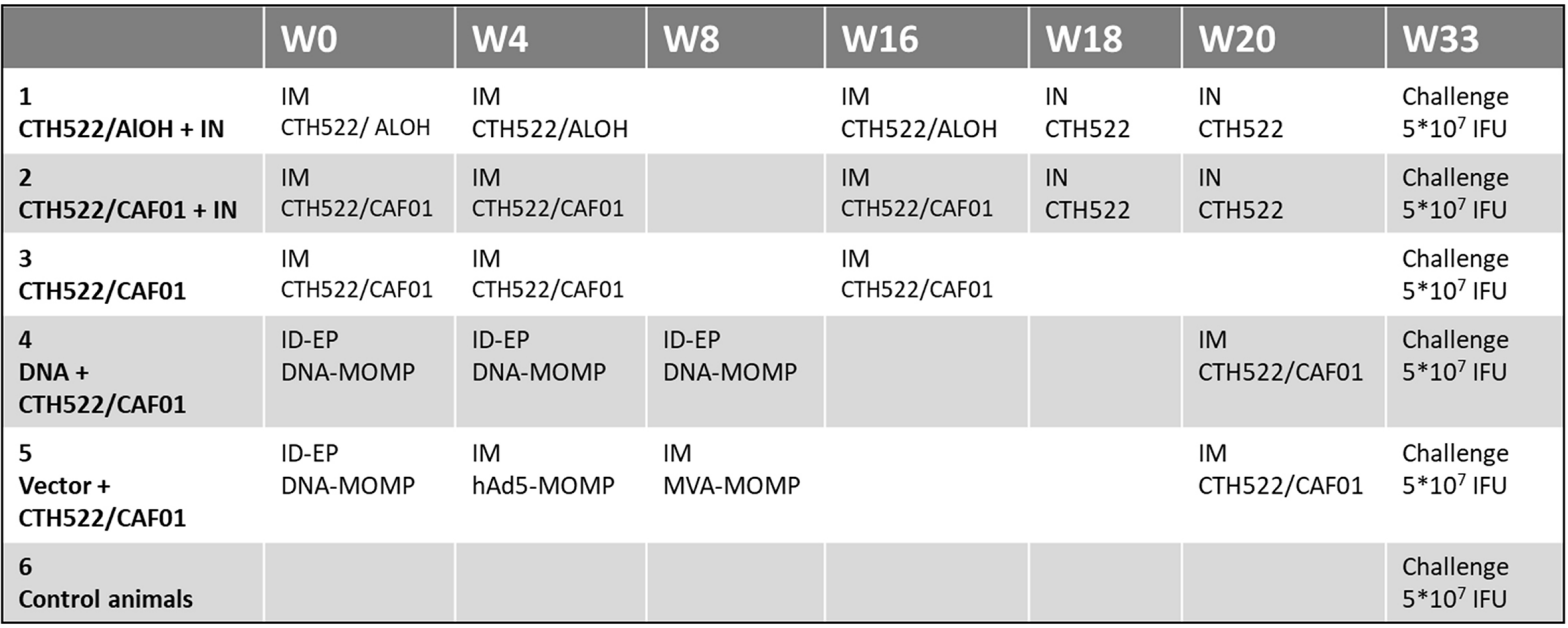
Study design. The figure shows the groups, the vaccines and the administration routes (intramuscular, IM, or intranasal, IN) used.

A total of 30 cynomolgus macaques were vaccinated following different prime-boost vaccination regimes including both protein, DNA and vector vaccines and different administration routes, as shown in **Figure 1** and **Supplementary Figures 6-10**). All animals remained healthy during the study and did not develop measurable adverse effects to the immunizations. In addition, we observed no significant fluctuations in the weight or temperature of the animals (**Supplementary Figures 1, 2**).

**Figure 2:**
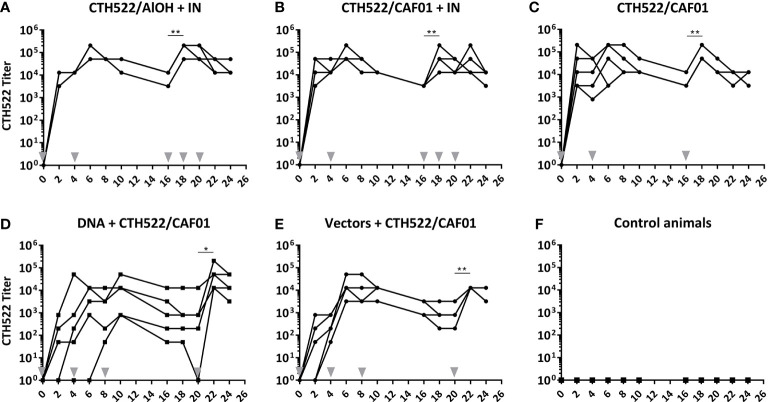
**(A–F)** Serum CTH522 IgG antibody responses. Cynomolgus macaques were immunized following different prime-boost regimes (*n = 5* per group) and serum was collected every second week. The specific CTH522 IgG antibodies in serum were evaluated by ELISA and expressed here as titer on a log10 scale. Grey arrowheads indicate vaccinations. Each line represents one animal. Statistical significance is indicated with asterisks *p < 0.05 and **p < 0.01.

At fixed time points after the immunizations, serum was collected and the levels of CTH522 specific IgG in serum were evaluated by ELISA. All 5 vaccinated groups established a significant serum CTH522 IgG response (**Figure 2**). However, the kinetics were slightly different in the 5 vaccinated groups. All animals in the CTH522/AlOH+IN, CTH522/CAF01 and CTH522/CAF01+IN groups raised a significant IgG titer after only 1 intramuscular (IM) immunization, and the IgG titer remained significant to the last sampling point (**Figures 2A-C**). The serum IgG levels increased slightly slower in the DNA+CTH522 and vectors+CTH522 groups, where all animals were seroconverted at week 8 and week 4, respectively (**Figures 2D, E**).

The CTH522/AlOH+IN, CTH522/CAF01+IN, and CTH522/CAF01 groups significantly increased serum IgG following the 3^rd^ IM booster vaccine in week 16 (**Figures 2A-C**). The DNA+CTH522 and vectors+CTH522 groups increased serum IgG levels significantly following the protein/CTH522/CAF01 IM booster in week 20 (**Figures 2D, E**). No significant differences were seen in the serum antibody responses following three times IM vaccinations with CTH522 formulated with either CAF01 or AlOH adjuvant (**Figures 2A, B**), and the intranasal (IN) non-adjuvanted booster immunizations in the CTH522/AlOH+IN and CTH522/CAF01+IN groups did not induce any significant increase in serum IgG antibody titers (**Figures 2A, B**).

The kinetics of the serum IgG response reflected the kinetics/levels in the vaginal fluids, indicating that the antibodies detected on vaginal surface were likely to be derived from the circulation. Similarly, the ocular IgG levels also followed the serum IgG levels and, as observed in the serum, showed that the IM booster in the DNA/vector groups increased the ocular IgG titer (**Supplementary Figure 3**).

### Serum antibody responses are directed against variable domains 1, 3 and 4 in the MOMP sequence

Further elucidation of the specific epitope recognition by the serum antibodies, following the different vaccination regimes, was next evaluated. Serum was analyzed at week 24, i.e. after the intranasal booster vaccines in group 1 and 2, and after the IM booster vaccines in the DNA and vector groups (group 4 and 5). These analyses were performed with a peptide array covering CTH522, consisting of 15 mer peptides with 14 amino acid overlap.

**Figure 3:**
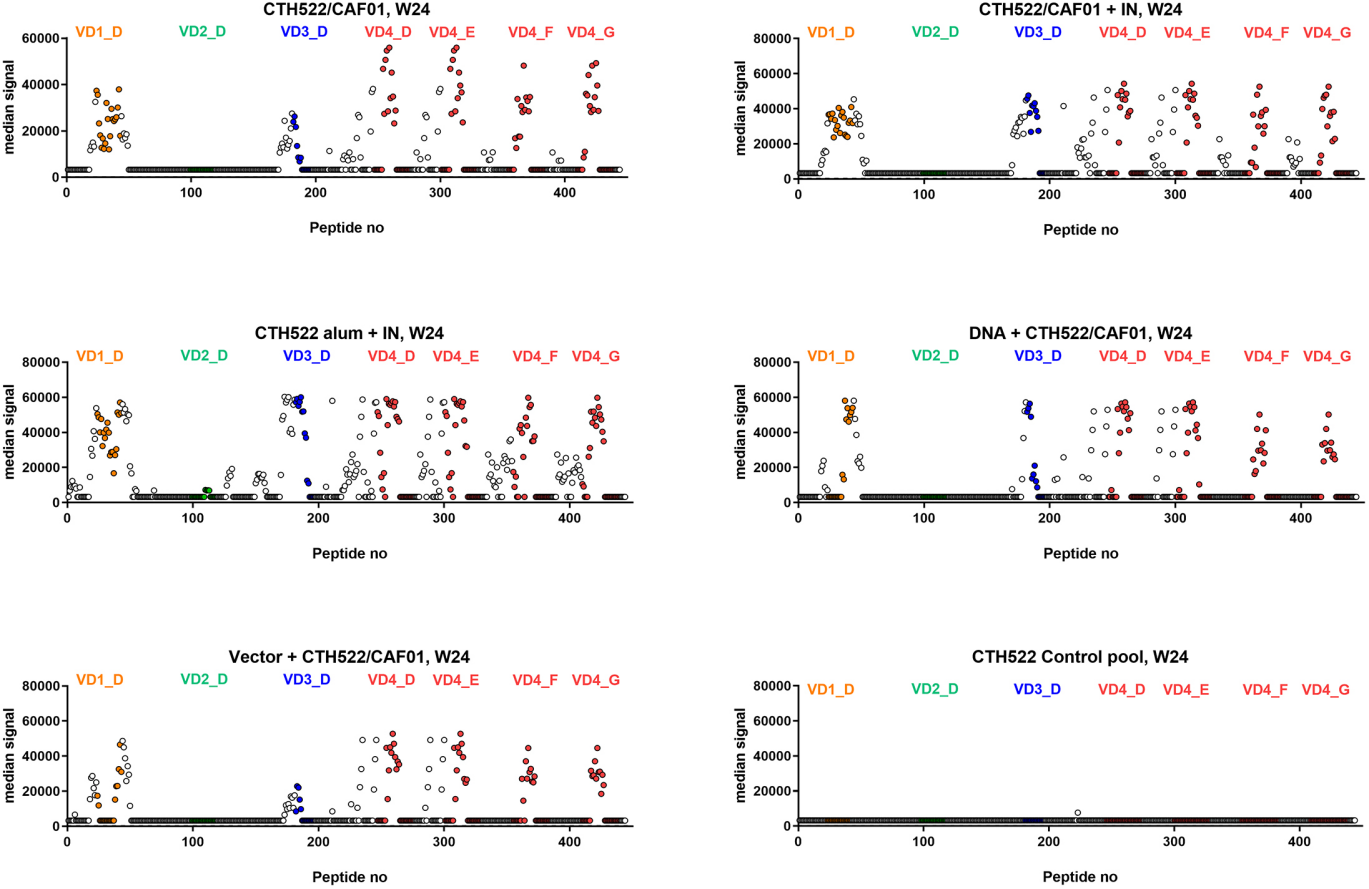
Serum epitope mapping at week 24. Cynomolgus macaques were immunized following different prime-boost regimes (n = 5 per group) and serum was collected at week 24. Peptide array IgG responses to CTH522 15mer overlapping peptides are presented as the median signal for each group with VD1 peptides (orange), VD2 (green), VD3 (blue) and VD4 from SvD, E, F and G (red) highlighted in colours.

Generally, the recognized peptides were located near and within the variable domains (VDs 1,3,4) of the MOMP. The CTH522/AlOH group even showed some recognition of the VD2 region. All animals established a strong response against the VD4 region that contains the conserved neutralizing VD4 epitope (11). The DNA group showed the narrowest recognition within the VDs (**Figure 3**), while the vector group showed a slightly broader recognition pattern. The CTH522/CAF01 and CTH522/AlOH groups showed the broadest recognition, especially against SvF and SvG.

### Serum antibodies were able to neutralize SvD, SvE, SvF *in vitro*

**Figure 4:**
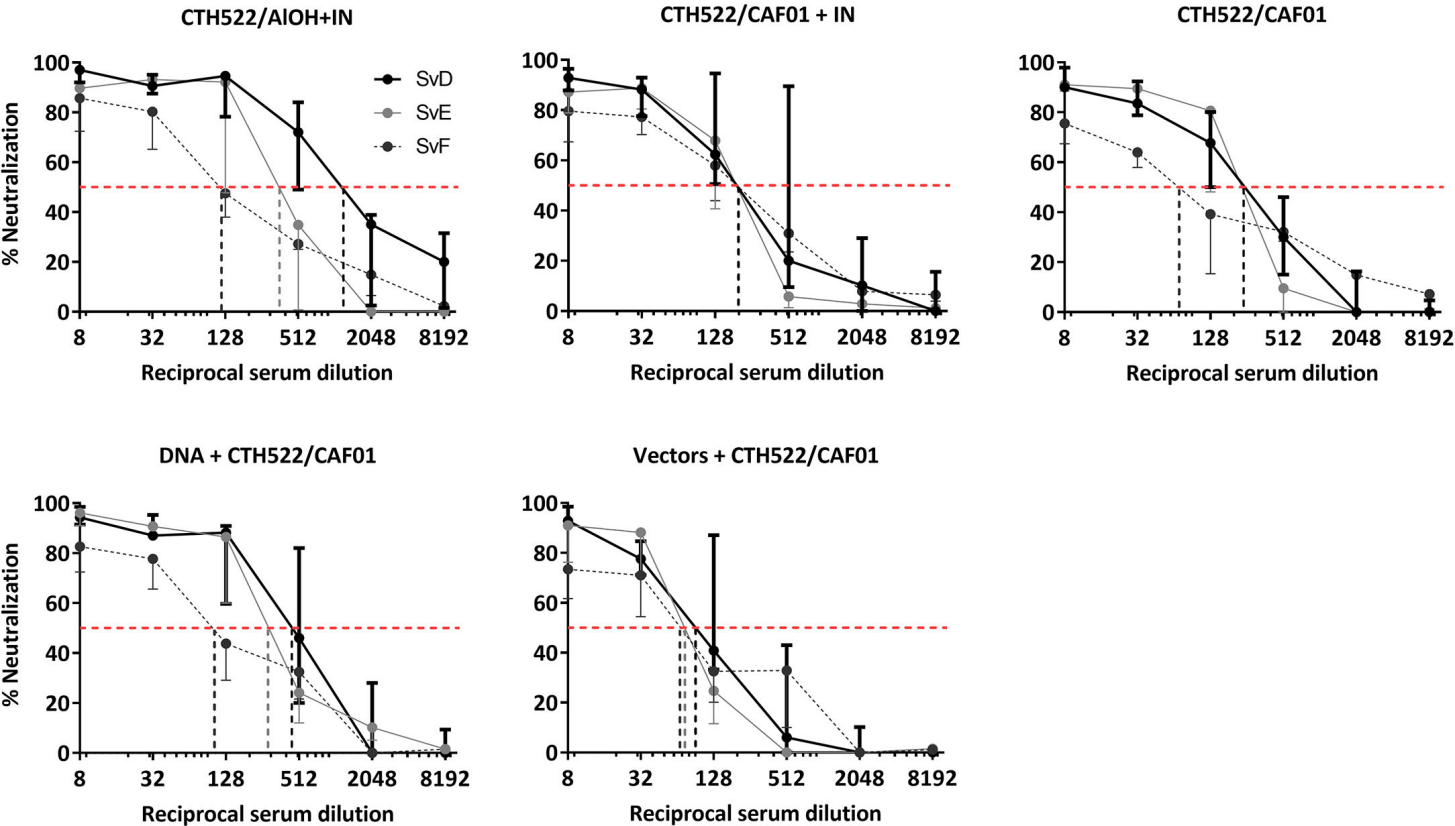
*In Vitro* serum neutralization of *C. trachomatis* SvD, SvE and SvF. Cynomolgus macaques were immunized following different prime-boost regimes (*n = 5* per group) and serum was collected after finishing the immunization schedule (week 24). *C. trachomatis* SvD, SvE and SvF was incubated with serum from each animal and the capacity of the serum antibodies to inhibit infection were evaluated by incubating the antibody-bacteria mixture on to HaK cells and staining for inclusions. The black line shows SvD, the grey line shows SvE and the dark grey dotted line shows SvF. The dotted red line indicates the reciprocal 50% neutralization titer for each serovar.

To evaluate the capacity of the vaccine induced antibodies to inhibit infection by *C. trachomatis* of its target cells, an *in vitro* neutralization assay was performed. Using this assay, we examined the neutralization of both SvD, SvE and SvF. Serum from week 24 was used for the assay, and serum from the naïve group was used as control. All five vaccinated groups showed a capacity to neutralize all three serovars (**Figure 4**). Against SvD the CTH522/AlOH+IN group showed the highest 50% neutralization titer, whereas against SvE the DNA/CTH522 group, closely followed by the CTH522/AlOH+IN group, showed the highest neutralizing titers. Even though all groups were able to neutralize SvF, the 50% neutralization titers were slightly lower for all groups against this serovar, compared to SvD and SvE. The vectors+CTH522 group showed the lowest 50% neutralization titers of all the vaccinated groups.

### Different vaccination regimes induced distinct T cell profiles

To evaluate the vaccination induced T cell responses, PBMCs were collected during the study period and cryopreserved. PBMCs from week 0/baseline and week 22 were re-stimulated with peptide pools (MOMP) and evaluated for intracellular cytokines by intracellular cytokine staining (ICS) and flow cytometry. The PBMCs were stained for the following cell markers: CD3, CD4, CD8, CD154, CD137 and cytokines: IFN-γ, IL-2, TNF-α, IL-22, IL-17A. The gating strategy is shown in **Supplementary Figures 4, 5**.

**Figure 5:**
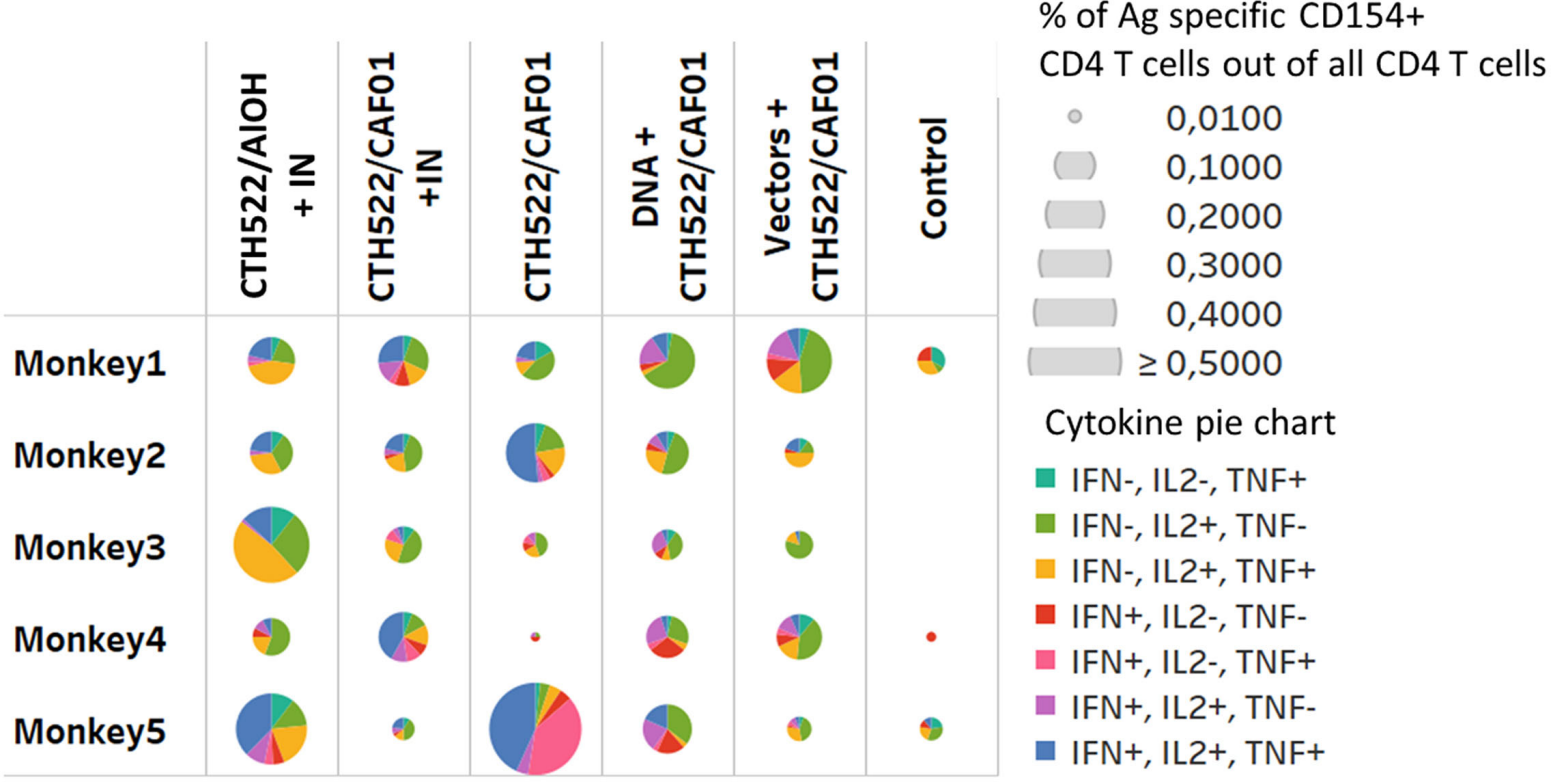
CD4 T cell responses. Multifunctional analyses week 22. Cytokine production by CD4^+^ T cells assayed by ICS. Multifunctional responses after stimulation with pools of overlapping peptides from MOMP are represented. The size of each pie is proportional to the percentage of CD154^+^CD4^+^ T cells expressing at least one cytokine, including IFN-γ, TNF-α, and IL-2, and the proportions of cells expressing IFN-γ and/or TNF-α and/or IL-2 cytokines are displayed in each pie.

**Figure 6:**
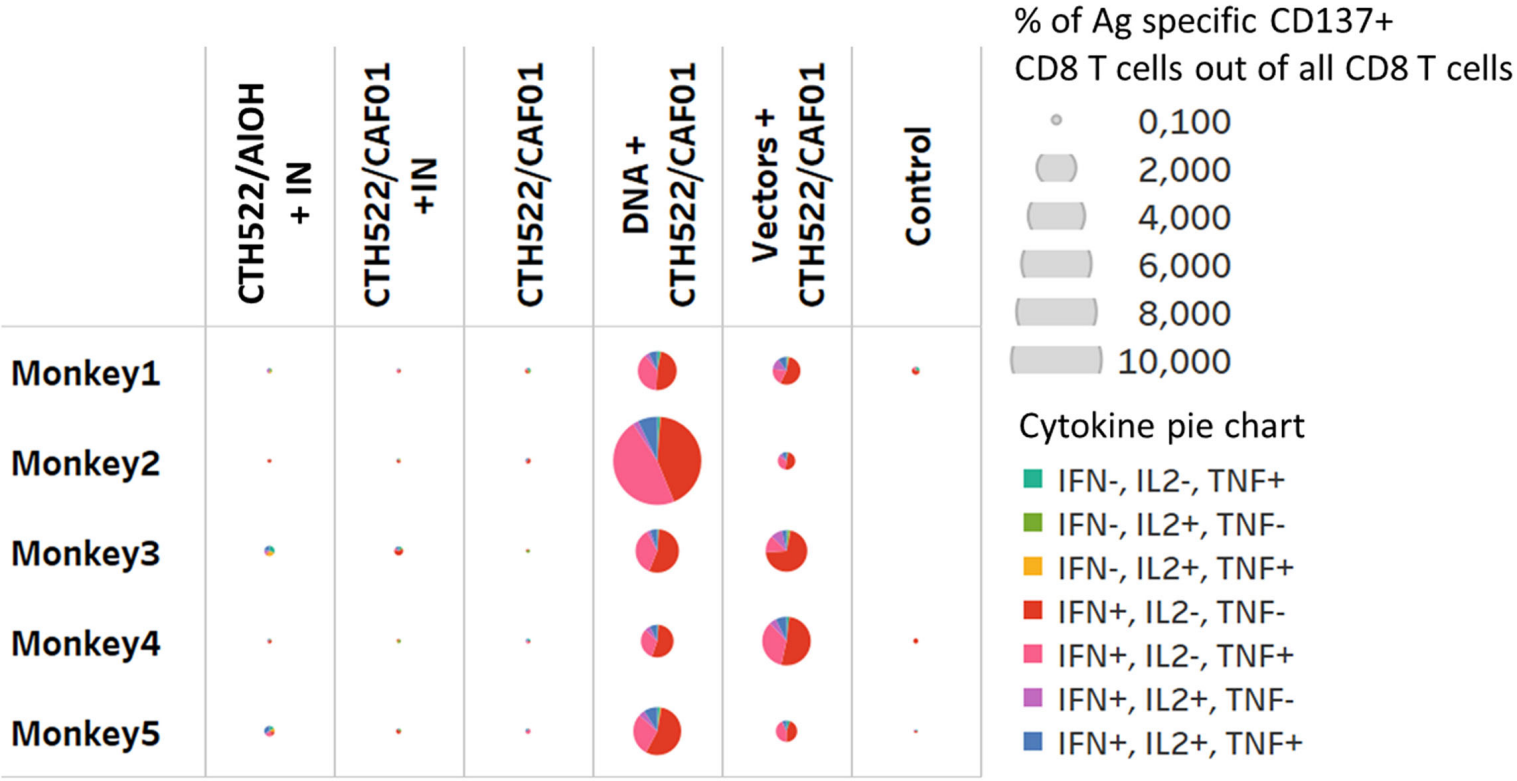
CD8 T cells responses. Multifunctional analyses week 22. Cytokine production by CD8^+^ T cells assayed by ICS. Multifunctional responses after stimulation with pools of overlapping peptides from MOMP are represented. The size of each pie is proportional to the percentage of CD137^+^CD8^+^ T cells expressing at least one cytokine, including IFN-γ, TNF-α, and IL-2, and the proportions of cells expressing IFN-γ and/or TNF-α and/or IL-2 cytokines are displayed in each pie.

Stimulation of PBMCs with the MOMP peptide pool induced a significant response. The percentage of antigen specific CD154 ^+^ CD4^+^ T cells expressing combinations of IL2, IFN-γ, and TNF-α were increased 2 weeks after the last immunization (W22) in all vaccinated groups (**Figure 5**). The majority of these T cells were multifunctional in the sense that they expressed several cytokines. The percentage of antigen specific CD137^+^ CD8 T cells producing cytokines were only increased in the DNA and Vector group (W22), (**Figure 6**, showing multifunctionality of CD137^+^ CD8^+^ T cells). In contrast to CD4^+^ T cells, which could be divided into 6 or more subsets based on their cytokine expression (**Figure 5**), the induced CD8 T cells were dominated by only three subsets. A larger proportion of the CD8 T cells produced only IFN-γ. The other dominant CD8 T cell subset produced both IFN-γ and TNF-α, whereas CD8 T cells producing all three cytokines (also the only CD8 T cell subset that expressed IL-2), represented the minor subset (**Figure 6**). No significant IL-17 or IL-22 responses were detected in the re-stimulated PBMCs (data not shown).

### Vaccination induced accelerated clearance of a vaginal *C. trachomatis* SvD infection

**Figure 7:**
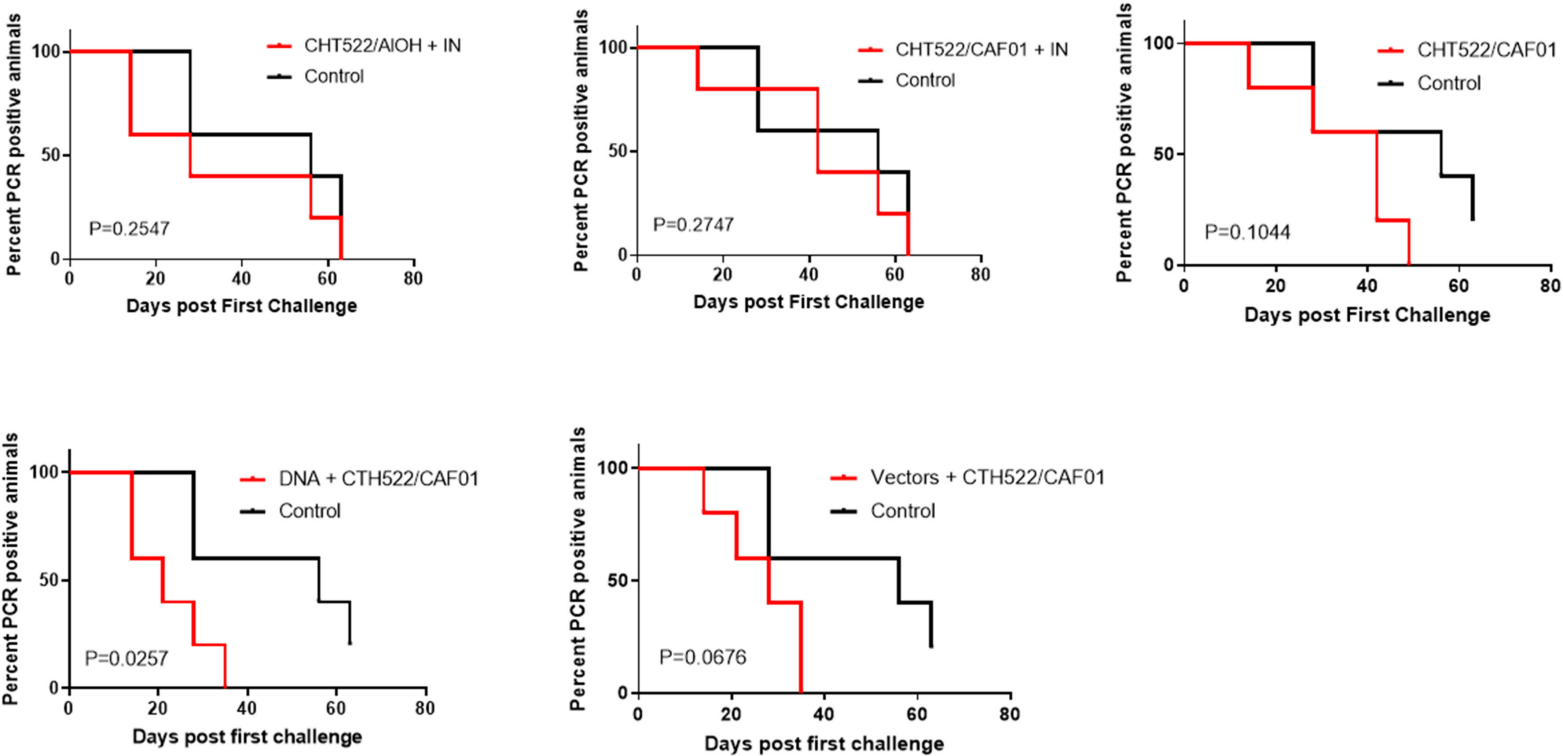
Clearance of vaginal *C. trachomatis* challenge infection Cynomolgus macaques were immunized following different prime-boost regimes (*n = 5* per group) and subsequently given a vagino-cervical challenge infection with 5*10^7^ IFUs *C. trachomatis* SvD. After the inoculation, vaginal swabs were collected for the following nine weeks. The vaginal chlamydial load was determined by qualitative PCR on swab material.

To evaluate protective efficacy of the vaccine-induced immune responses, the animals were challenged with a vaginal infection with 5x10^7^ IFUs *C. trachomatis* SvD. Following inoculation, the vaginal chlamydial load was determined by PCR detection of chlamydial DNA in vaginal swabs (**Figure 7**). The infection was cleared in all animals by week 5 in the DNA+CTH522/CAF01 group and the Vector+CTH522 group, by week 6 in the CAF01/CTH522 group and by week 8 in the CTH522/Alum+IN group (**Figure 7**). In the control group, at week 8, 80% of the animals had cleared the infection and 1 animal remained PCR positive at week 9. The IN booster in the CTH522/CAF01+IN group did not increase the protection (**Figure 7**). A Log-rank (Mantel-Cox) test, comparing all groups showed a P value of 0,0994, and by comparing each of the groups individually against the control groups, only the DNA-CTH522 group showed a P value below 0.05 (P value=0.0257).

## Discussion

The present study was designed to induce cellular immunity (CD4 and CD8) combined with humoral immunity and to tailor the phenotype of adaptive immunity with MOMP based Chlamydia vaccines. We evaluate immunogenicity and protective efficacy of combined multi-component immunization strategies with both protein vaccines (CTH522, Con E), DNA vaccine and recombinant viral vector (MVA-MOMP), and adeno vector based vaccines, in a non-human primate (NHP) model.

The data showed that all immunization regimes were immunogenic and induced both a cellular and a humoral response consisting of neutralizing antibodies (**Figures 2**-**4**). The protein vaccine CTH522 formulated with either CAF01 or AlOH showed a fast induction of systemic antigen-specific IgG (**Figure 2**). We did not observe any significant differences between the responses induced by AlOH and CAF01 adjuvanted vaccines, which is in agreement with the responses observed in humans with these adjuvants (16). The DNA and vector vaccination regimes also induced significant titers of antigen-specific systemic antibodies, which markedly increased following the protein booster immunization (**Figure 2**). In agreement with our results, a previous study in mice also showed that protein vaccination regimes induced higher antigen-specific antibody levels compared to DNA and vector vaccine antigens and also efficiently boosted titers for these groups (15).

MOMP contains four VD’s that are exposed on the surface of the infectious form of *C.t.* These regions are main target of antibody-dependent immunity, both through neutralizing antibodies and Fc receptor mediated immunity (18). Previous studies have shown that adoptive transfer of vaccine-induced neutralizing serum markedly reduced early bacterial shedding following a *C. trachomatis* infection (6, 11, 19–21). Thus, antibodies seem to play a role in neutralizing/controlling the initial inoculum, probably in cooperation with cellular immunity. By detailed B cell epitope mapping we found recognition of VD regions 1, 3, 4 in all vaccine groups. The DNA and vector groups also recognized the VD1, 3 and 4 regions, although these groups showed a slightly narrower recognition within these VDs compared to the CAF01 and AlOH groups, probably reflecting that the Con E antigen in the vector groups does not contain the VD4 region of SvF and G (**Figure 3**). Recognition of the VD4 region correlated with the ability of all vaccine strategies to induce neutralizing antibodies against SvD, SvE and SvF.

Parenteral immunization with CTH522 in CAF01 and AlOH induced significant levels of mucosal antibodies (IgG) in the genital tract and the eye and levels correlated with serum titers (**Supplementary Figure 3**, week 18). DNA and vector vaccines alone did not induce mucosal IgG, which reflects the lower serum titers compared to protein alone. However, following the CTH522/CAF01 IM booster, these groups significant increase in serum Ab. levels and of both vaginal and ocular IgG, at par with the protein alone (**Supplementary Figure 3**, week 18). The parallel assessment of CTH522 in the different priming adjuvant systems (AlOH and CAF01) followed by two times intranasal boosting with antigen had no boosting effect on mucosal antibody levels (**Supplementary Figure 3**, week 22). Thus overall serum IgG levels are reflected on the mucosa for all groups. A recent human phase-I trial also evaluated the parallel assessment of CTH522 in AlOH and CAF01, followed by two times intranasal boosting. Here mucosal IgG titers also correlated well with serum IgG concentrations (16). However, in contrast to the findings in the NHP model, the human trial showed effect of IN boosting in the CAF01 group, resulting in elevated levels of both IgG and IgA. Unfortunately, IgA evaluation was not possible in the current study, due to limited material and false positive signals resulting from a cross reacting secondary antibody used in the assay. Both studies used the VaxINator device, which is designed to atomize nasal medications for mucosal adsorption. However, the nasal cavity of humans is much more spacious than in NHP’s and it could be speculated that this could influence the evenly antigen distribution and uptake.

Several studies in both humans and rodents have demonstrated the importance of Th1 T cell immunity and the effector cytokine IFN-γ (8, 22–27). In contrast, less is known about the protective role for CD8 T cells (28–33). All the vaccine groups induced CD4 T cells, but only the DNA and Vector group showed a CD8 T cell response (**Figures 6** and **7**). Based on the cytokines measured, the CD8 T cells were composed of a limited number of subsets compared the CD4 T cells, which could be divided into 6 or more subsets, most of which produced several cytokines (**Figure 7**). CD4 T cells showed more IL-2 expressing subsets than CD8 T cells, which primarily included only one IL-2 expressing subset, the one that expressed IL-2 together with IFN-γ and TNF-α (**Figure 6**). CD8 T cells were dominated by a large population producing only IFN-γ, whereas the other dominant CD8 T cell subset produced both IFN-γ and TNF-α. This cytokine pattern among CD4 T cells and CD8 T cells is not unlike what has been observed in mice, when testing vaccine strategies able to induce both CD4 and CD8 T cells (34).

To evaluate protective efficacy of the vaccine-induced immune responses, the animals were challenged with a vaginal infection with 5*10^7^ IFUs *C. trachomatis* SvD. Following inoculation, protection, measured by the percentage of animals able to clear the infection, was observed in the CAF01/CTH522, DNA prime CTH522/CAF01 boost and in the vector group. In particular, in the DNA group (boosted with CAF01/CTH522) all animals had cleared the infection by week 5, in contrast to control animals where only 40% had cleared the infection. The DNA prime/CTH522/CAF01 boost group had the overall most multifaceted immune response with high level of neutralizing Abs combined with strong CD4 and CD8 T cell responses (**Figures 4**-**6**). One question, which will be important to address in future studies is if the added effect of CD8 T cells could also be achieved by a CD4 T cell inducing vaccine strategy with the ability to induce even higher levels of IFNγ/TNFα in CD4 T cells, or if the added effect in the DNA group is due to unique CD8 T cell effector functions.

A similar strategy involving DNA vaccination followed by boosting with immune-stimulating complexes (ISCOM) of MOMP protein (35) induced strong protection against *Chlamydia muridarum* lung infection. In that study DNA-MOMP (or MOMP/ISCOMS) induced significantly less protection (35). Another study showed that vaccination of mice with DNA MOMP plasmids, without a subunit vaccine, failed to protect against a genital *C. trachomatis* challenge (36), indicating that the DNA strategy benefits from being supplemented with a subunit vaccine inducing CD4 T cells and antibodies. It should be noted that DNA vaccines have shown relatively low immunogenicity profiles in human clinical trials, which may have to be addressed in future studies (37).

Although the *C.t*. infection dose used in this study is higher than the dose experienced by a natural infection in humans, it was selected to ensure that all animals were infected, which is important considering the small number of animals in each group. The dose is in line with the dose used in previous studies, which ranged from 10^6^ to 10^8^ (38, 39). Lowering the dose in our model led to increased variation regarding infection take (data not shown). Lower doses given repeatedly has also been used (40, 41). However, this is not an optimal protocol when the objective is to test the protective efficacy of vaccines, especially if natural immunity is mediating protection before the infection protocol has been completed. It can be speculated that using a high dose may require an immune response that contains all the arms of the immune system, such as antigen specific CD4 T cells, CD8 T cells as well as neutralizing antibodies, in order to be able reduce the bacterial levels. That would also explain why the neutralizing antibodies induced in all the groups did not lead to significant protection in all the groups [although we did notice a trend to early protection in most groups, where antibodies are thought to play a role (**Figure 7**)]. We are presently trying to develop a more physically relevant model.

Taken together, our data showed that the CTH522 antigen formulated in the adjuvant CAF01 or AlOH can induce both multifunctional T cells and neutralizing antibodies in NHPs. Moreover, our results suggested that a heterologous vaccine strategy consisting of a DNA vaccine and a CTH522/CAF01 subunit vaccine, represents a promising vaccine strategy for induction of a multifaceted immune response with high level of neutralizing Abs combined with strong CD4 and CD8 T cell responses that can significantly accelerate clearance of a *C. trachomatis* infection.

## Materials and methods

### Vaccine antigens and adjuvants

Different vaccine antigens and adjuvants were used in this study: CTH522 vaccine antigen, based on MOMP (11) was administered through the intramuscular route (IM) with CAF01 adjuvant (42) or Aluminium hydroxide (AlOH) adjuvant, or through the intranasal route (IN) without adjuvant. Plasmid DNA vaccine, pcDNA3.1-MOMP, was made by cloning consensus MOMP into the plasmid vector pcDNA3.1 (14) and administrated by intra-dermal (ID) route with electroporation (EP) in the back. Recombinant Human Adenovirus serotype 5 (rHuAd5) vaccine expressing MOMP (HuAd5-MOMP) was made by homologously recombining consensus MOMP (AAC45154.1) into an E1 and E2 deleted HuAd5 plasmid pAL1112 vector and administered through intramuscular route (14). Modified vaccinia Ankara-MOMP vaccine made by recombining consensus MOMP (AAC45154.1) into the p3186 plasmid and attenuated MVA vector (14), and administered through intramuscular route.

### *C. trachomatis* serovar D stock

*Chlamydia trachomatis* serovar D (*Ct* SvD; UW-3/Cx, ATCC^®^ VR-885™) was obtained from American Type Culture Collection (ATCC, Masassas, VA) and propagated in HeLa-229 cells, harvested, and purified as described by Caldwell et al. (43). The *C. trachomatis* SvD stock was stored at -80°C in 0.2 M sucrose, 20 mM sodium phosphate (pH 7.4), and 5 mM glutamic acid (SPG) and the concentration of inclusions forming units (IFUs) were determined in HeLa-229 cells and McCoy cells.

### Non-human primates

Thirty cynomolgus macaques (*Macaca fascicularis*) weighing 3 to 5 kg were included in the study handled in biosafety level 3 (ABSL3) NHP facilities of IDMIT (“Infectious Disease Models and Innovative Therapies” at the CEA “Commissariat à l’Energie Atomique,” Fontenay-aux-Roses, France; accreditation no. #D92-032-02). The protocols were approved by the institutional ethical committee “Comité d’Ethique en Expérimentation Animale du Commissariat à l’Energie Atomique et aux Energies Alternatives” (CEtEA #44) under statement number A14-026. The study was authorized by the “Research, Innovation and Education Ministry” under registration number APAFIS#720-201505281237660. All animals were tested negative for SIV, SHIV, STLV (simian T-lymphotropic virus), herpes B virus, filovirus, SRV-1, SRV-2 before the study and confirmed to be seronegative for *Chlamydia* infection. All experimental procedures were conducted according to European guidelines for animal care (“Journal Officiel de l’Union Européenne”, directive 2010/63/UE, September 22, 2010).

### Vaccination protocols

The 30 macaques were randomly divided into 6 groups with 5 animals in each. The different vaccination prime-boost regimes for the 6 groups are illustrated in detail in **Figure 1**. During all vaccinations the macaques were sedated with ketamine chlorhydrate (10 mg/kg body weight inramuscularly). CTH522 (85 µg per animal) was administered by the intramuscular (IM) route with either CAF01 adjuvant (625 µg/125 µg) or AlOH adjuvant (0.425 mg), as shown in **Figure 1**. IM immunizations were performed in 0.6 ml volume in the right thigh (*M. quadriceps*) of the animal. CTH522 Intranasal (IN) immunizations (30 µg per animal) were performed with a Vaxinator device connected to a 1 ml syringe with two successive administrations of 0.25 ml, one in each nostril. The DNA-MOMP vaccine was administered by intradermal (ID) route with electroporation (EP) in the skin of the back. 6 repeated volumes of 0.1 ml were injected ID (in total 1 mg) followed by EP using the Nepagen™ system (settings: 6 square-ware pulses of 10 ms with 90 ms intervals at 110V, (300-700mA)). The hAd5-MOMP vaccine was administered IM with a dose of 10^11^ viral particles in 1 ml per animal. The MVA-MOMP vaccine was administered IM with a dose of 4*10^8^ PFU per animal in 0.5 ml volume **. The naïve group was not given any vaccination, and was considered to be the control group.

### Sample collection

During all sampling procedures the macaques were sedated with ketamine chlorhydrate (10 mg/kg body weight IM). Blood was collected *via* femoral venipuncture at week 0, 2, 4, 6, 8, 10, 16, 18, 20, 22, 24. Cervico-vaginal fluid for antibody detection, was collected with Weck-Cel^®^spears (Medtronic Ophtalmics, Jacksonville, FL, USA) at week 4, 8, 16, 18, 20, 22 and 24. Cervico-vaginal samples for chlamydial load detection were collected with regular flock swabs (Mast Diagnostic - ref.: 552C/10). Following contact with the vagino-cervical mucosa, the swab was placed in a vial containing UTM-RT medium (Mast Diagnostic – ref.: 330C/6) and kept on ice.

### Vaginal challenge infection with 5*10^7^ IFUs of *C. trachomatis* SvD

The animals were sedated with ketamine chlorhydrate (10 mg/kg IM), placed in ventral recumbency with their hips elevated and 1 ml of the inoculum was atraumatically applied directly to the vaginal mucosa at the vagino-cervical transition using a 1 ml syringe. A dose of 5*10^7^ IFUs were given to each animal. The animal was allowed to stay in ventral recumbency with their hips elevated for a short time after the inoculation.

### Enzyme-linked immunosorbent assay

CTH522 specific antibodies in serum and swab samples were detected with an indirect ELISA. Maxisorp^®^ plates (NUNC A/S, Roskilde, Denmark) were coated with CTH522 (0.5 µg/ml) over night at 4°C. The isotypes IgG and IgA were detected with HRP-conjugated antibodies specific against non-human primate IgG (43R-IG020HRP, Fitzgerald, Acton, MA, USA, 1:75.000) and IgA (43C-CB1631, Fitzgerald, USA, 1:50.000). The reactions were visualized with TMB PLUS substrate (KemEnTec, Taastrup, Denmark) and stopped with 0.5 M sulphuric acid. The plates were read on an ELISA reader at 450 nm with correction at 650 nm. A positive control (serum from a previous study) was used as an internal standard to correct for plate-to-plate variation. Two wells were run without substrate as a negative control on each plate. The antibody titers were calculated as the reciprocal of the highest dilution with an optical density (OD) value higher than the cut-off. The cut-off was determined from the day 0 sample mean + 2*SD.

### Peptide array and analysis

The CTH522 peptide array was designed with triplicates of immobilized 15mer peptides overlapping by 14 amino acids which were printed on functionalized glass slides and incubated with serum samples by JPT Peptide Technologies, Berlin. Briefly, the array was incubated with serum samples collected at week 24 and IgG peptide complexes were visualized using biotinylated goat anti–monkey IgG and Alexa Fluor^®^ 647 Streptavidin. Slides were scanned with a high-resolution scanner at 635 nm and data reported as arbitrary fluorescence units. The mean of triplicate values was determined, if the coefficient of variation was larger than 0.5, only the 2 closest values were used. 10% of the maximum intensity signal (65,535) was used as cut-off for a positive response, and mean signals below this threshold were assigned a value corresponding to half the threshold value (3277).

### *In vitro* neutralization assay

The *in vitro* neutralization assay was performed to test the vaccine-induced serum antibodies’ capacity to neutralize *Chlamydia* infection of HaK cells *in vitro*. The assay principally followed the protocol previously described (44). First, serum samples from individual animals were incubated at 56°C for 30 minutes to inactivate complement. *Chlamydia* stock (SvD, SvE and SvF) was mixed and incubated with serial dilutions of inactivated serum from each of the vaccinated animals for 30 minutes and then inoculated onto a monolayer of HaK cells in 96-well flat-bottom plates in duplicates. Following 30 hours of incubation, cells and inclusions were fixated and visualized with polyclonal rabbit anti-Ct043 serum and fluorescence labeled secondary antibody (Alexa Flour 488, goat-anti-rabbit IgG, A11008, Life Technologies) in the dilution 1:500. Counting of inclusions was performed manually in 20 fields of view at 40x magnification (Olympus IX71 inverted fluorescence microscope). Neutralization was calculated as the percentage reduction in the mean number of IFUs compared to a pool of sera from control group animals (naïve).

### Intracellular PBMC cytokine staining and flow cytometry

PBMCs were isolated and (1-2 x 10^6^) were resuspended in 150 µl of complete medium containing 0.2 µg of each costimulatory antibody CD28 and CD49b. Stimulation was performed in 96 well/plates using 2 µM of each peptide pool or SEB (as positive control) or medium alone (as negative control). Brefeldin A was added to each well at a final concentration of 10 µg/ml and the plate was incubated at 37°C, 5% CO_2_ overnight. The cells were then washed, stained with a viability dye (violet, fluorescent reactive dye, Invitrogen), fixed and permeabilized with the BD Cytofix/Cytoperm reagent. Permeabilized cell samples were then stored at -80°C before the staining procedure. Antibody staining was performed in a single step following permeabilization. All the used antibodies are listed in **Table 1**. After 30 min of incubation on ice in the dark, cells were washed in BD Perm/Wash buffer. Cells were counted with an LSR II (BD) immediately after the staining procedure and FlowJo software was used for data analysis.

**Table 1:** Antibodies used for staining of immune cell markers and intracellular cytokines.

	FLUOCHROME	CLONE	FUNCTION
BLUEVID	Blue fluorescent dye	n.a.	Dead/live cells
CD3	APC-Cy7	SP34-2	T cells
CD4	V500	L200	CD4 T cells
CD8	PE-Cy7	RPA-T8	CD8 T cells
CD154	FITC	TRAP-1	Ag specific marker for CD4 T cells
CD137	APC	4B4-1	Ag specific marker for CD8 T cells
IFN-γ	V450	B27	Cytokines Th1
IL-2	PerCP-Cy5.5	MQ1-17H12	Cytokines Th1
TFN-α	BV605	Mab11	Cytokines Th1/Th17
IL-22	PE	2G12A41	Cytokines Th1
IL-17A	AF700	N49-653	Cytokines Th17

n.a., Not applicable.

### Detection of vaginal chlamydial DNA by qualitative PCR

The Abbott RealTime CT/NG assay (ABBOTT, Rungis, France) is an *in vitro* polymerase chain reaction (PCR) assay for the direct, qualitative detection of the plasmid DNA for CT (*C.trachomatis*) and NG (*N. gonorrhoeae*) genomes. Nucleic acid were extracted from UTM (universal transport media) samples on M2000SP and amplification were performed on M2000SP, according to the manufacturer’s instructions. Results were expressed as cycle threshold (Ct).

### Statistical analysis

All statistics were performed with GraphPad Prism software (version 8.1.2). The not-normally distributed data were analyzed with non-parametric tests; Kruskal–Wallis test and Dunn’s multiple comparison test to determine the significance of the difference between groups. Curves showing percent PCR negative animals were compared with a Log-rank (Mantel-Cox) statistical test. The comparisons were considered statistical significant if the *P* value was < 0.05 (*P* < 0.05). Further levels of significance are indicated with asterisks *P < 0.05, **P < 0.01, ***P < 0.001.

## Data availability statement

The original contributions presented in the study are included in the article/**Supplementary Material**. Further inquiries can be directed to the corresponding author.

## Ethics statement

The animal study was reviewed and approved by “Comité d’Ethique en Expérimentation Animale du Commissariat à l’Energie Atomique et aux Energies Alternatives” (CEtEA #44) under statement number A14-026. The study was authorized by the “Research, Innovation and Education Ministry” under registration number APAFIS#720-201505281237660.

## Author contributions

Conceived and designed experiments: JD, FF, EL, PA, RG, RS. Performed experiments: VC, DD, BD, SL, CD, CJ, ND-B, CB, BB, AT. Produced vaccines and antigen constructs: IR, PM, AO. Analyzed the data: JD, EL, FF, IR, HJ. Drafted and edited the paper: JD, EL, FF. Each of the listed co-authors made substantial contributions to the work through design and conception, and/or acquisition, analysis, and interpretation of the data. All authors contributed to the article and approved the submitted version.

## Funding

This study was supported by the European Commission through the ADITEC consortium contract (FP7-HEALTH-2011.1.4-4-280873) and The Innovation Fund Denmark (069-2011-1). The Infectious Disease Models and Innovative Therapies research infrastructure (IDMIT) is supported by the Domaine d’Intérêt Majeur (DIM) “One Health” program, and the “Programme Investissements d’Avenir” (PIA), managed by the ANR under references ANR-11-INBS-0008 and ANR-10-EQPX-02-01.

## Acknowledgments

We thank the Fondation Dormeur, Vaduz for the donation to Viral Evolution and Transmission Unit for laboratory instruments relevant to this project.

## Conflict of interest

FF, IR, PA and AWO are co-inventors on a patent application on vaccines against chlamydia [WO2014146663A1]. All rights have been assigned to Statens Serum Institut, a Danish not-for-profit governmental institute.

The remaining authors declare that the research was conducted in the absence of any commercial or financial relationships that could be construed as a potential conflict of interest.

## Publisher’s note

All claims expressed in this article are solely those of the authors and do not necessarily represent those of their affiliated organizations, or those of the publisher, the editors and the reviewers. Any product that may be evaluated in this article, or claim that may be made by its manufacturer, is not guaranteed or endorsed by the publisher.
