# Supplementary material for: Multi-component prime-boost Chlamydia trachomatis vaccination regimes induce antibody and T cell responses and accelerate clearance of infection in a non-human primate model
Source: Front Immunol. 2022 Nov 23;13:1057375. doi: 10.3389/fimmu.2022.1057375 (PMC9726737; doi:10.3389/fimmu.2022.1057375)
Supplement: Supplementary file 1 [file DataSheet_1.pdf]

Supplemental information

Multi-component prime-boost *Chlamydia trachomatis* vaccination regimes induce antibody and T cell responses and accelerate clearance of infection in a non-human primate model

Emma Lorenzen, Vanessa Contreras, Anja W. Olsen, Peter Andersen, Delphine Desjardins, Ida Rosenkrands, Helene Bæk Juel, Delache Benoit, Langlois Sebastien, Delaugerre Constance, Joubert Christophe, Dereuddre-Bosquet Nathalie, Bébéar Cécile, De Barbeyrac Bertille, Arabella Touati, Paul F McKay, Robin J. Shattock, Roger Le Grand, Frank Follmann and Jes Dietrich

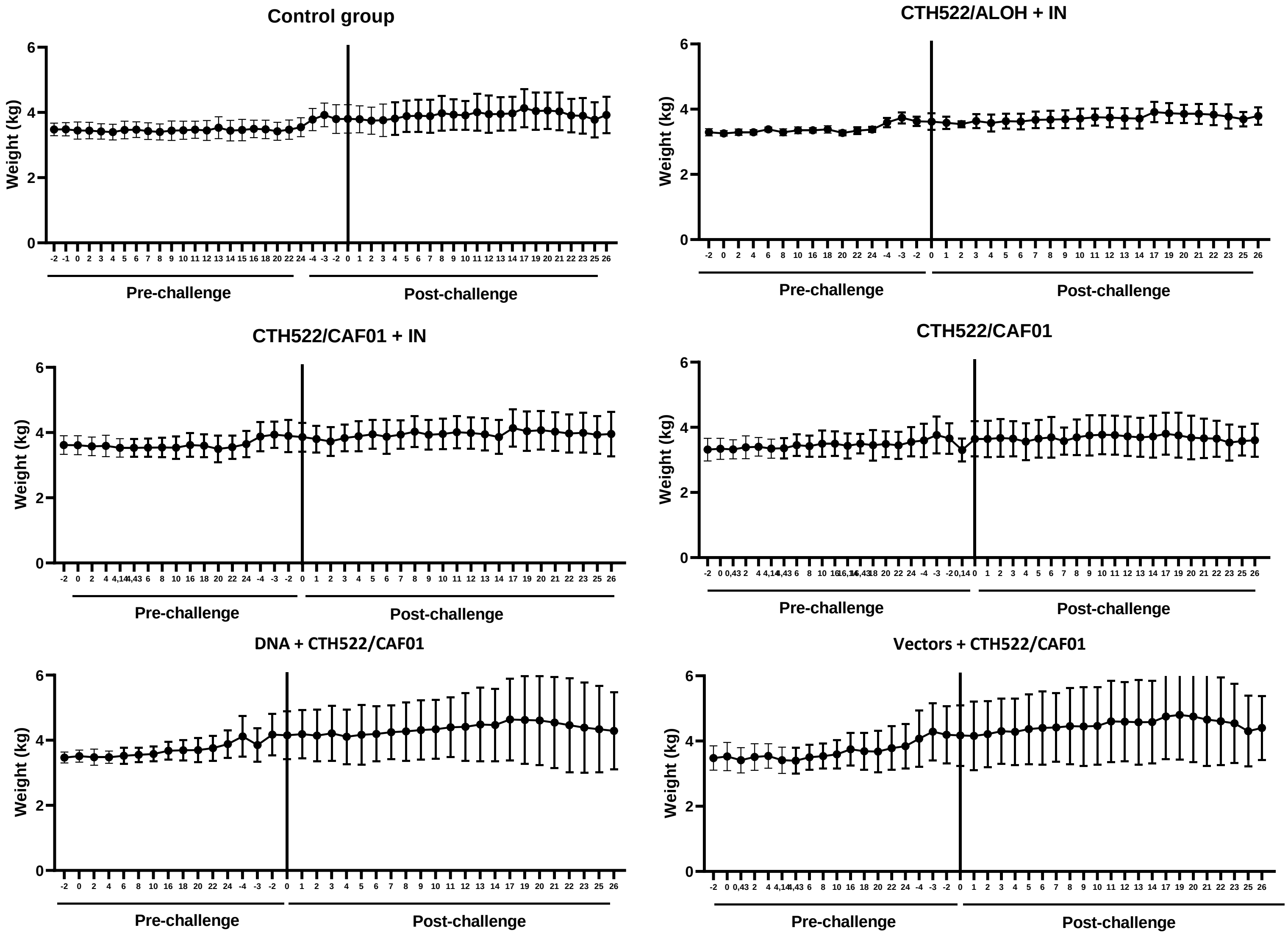

Supplementary figure 1. Weight curves pre and post challenge

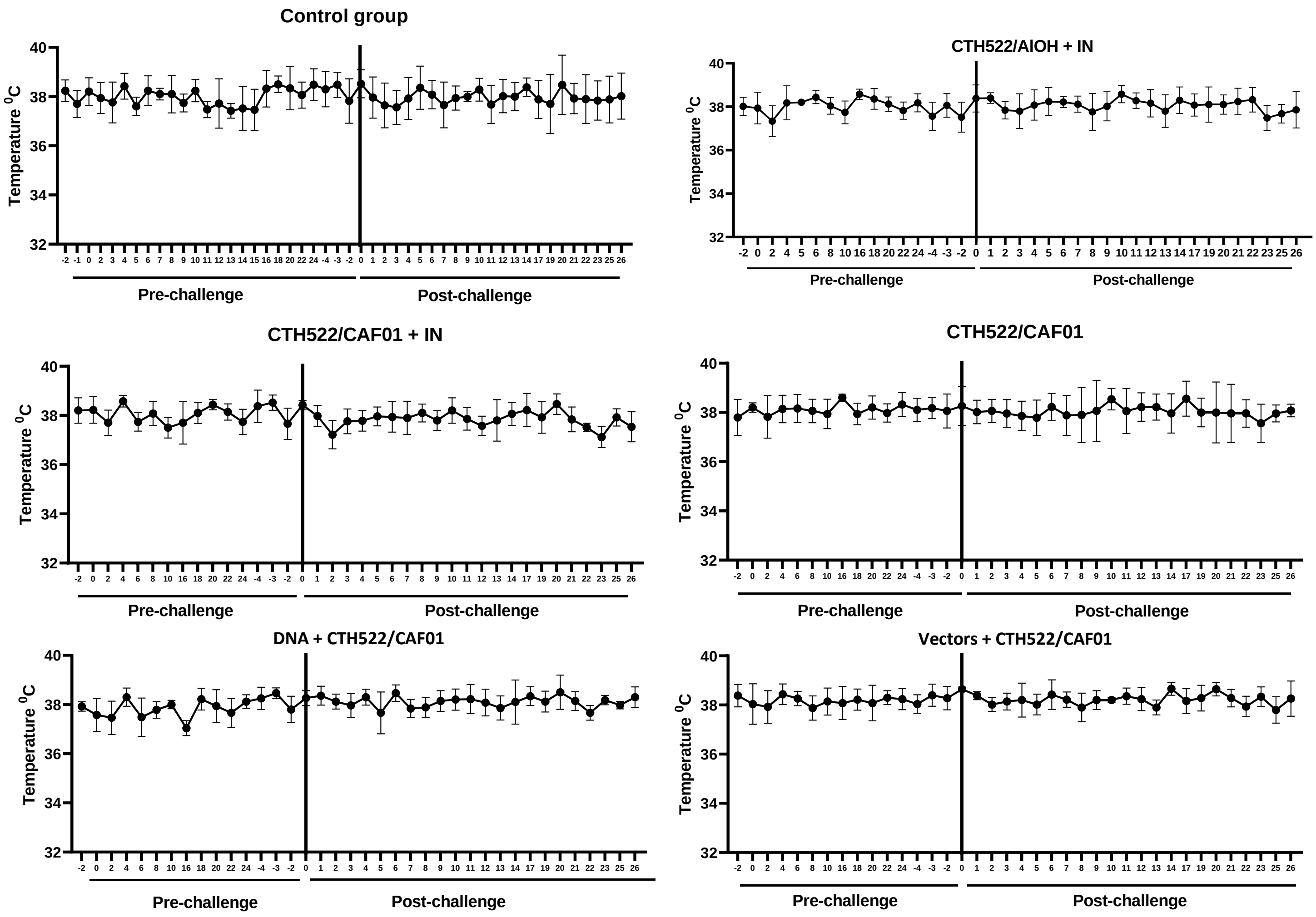

Supplementary figure 2. Temperature pre and post challenge

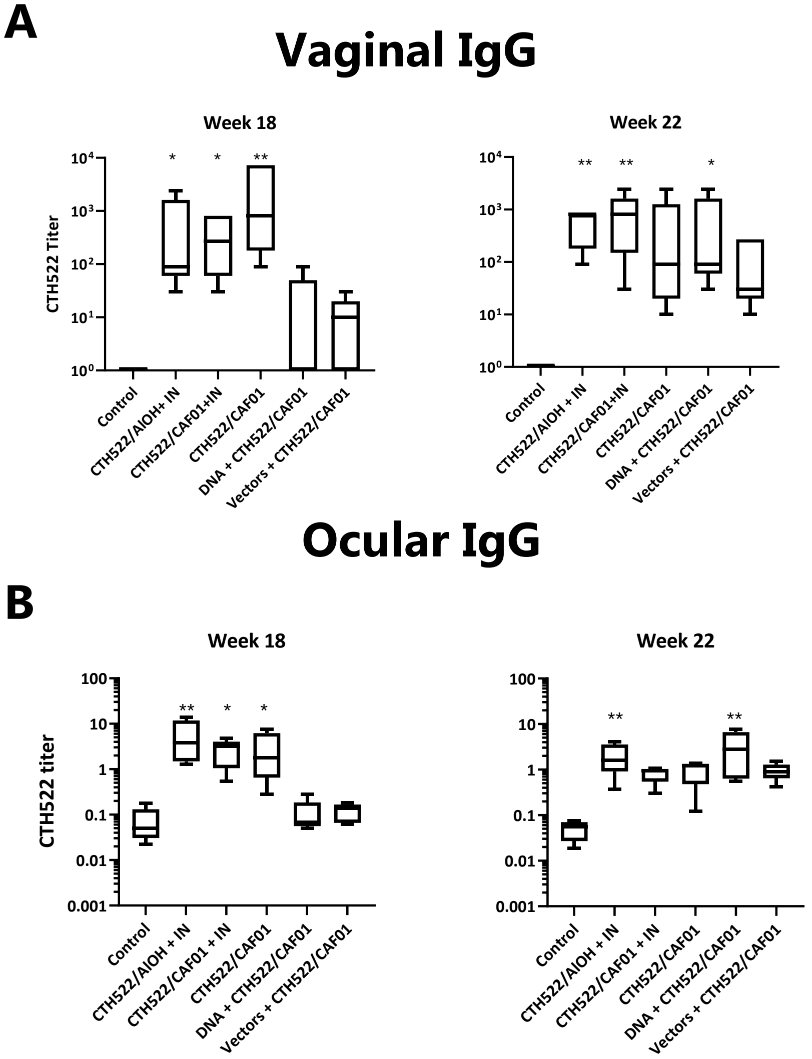

Cynomolgus macaques were immunized following different prime-boost regimes ($n = 5$ per group) and vaginal fluid was collected with Weck-cel spears. The specific CTH522 IgG levels in the vaginal fluid were determined at week 18 and 22 by ELISA, and expressed here as titer (µg/ml) on a log10 scale. Tears were collected with a Schirmer strip inserted into the external lower conjunctival sac. After 2 minutes, the strip was removed and placed in a 0.5 ml tube punctured in the center of the bottom, which again was placed in a larger tube (1.5 ml) and kept on ice until centrifugation for 5 min at maximum (22700 g). The tear fluid was stored at -20 °C until antibody quantification.

Statistical significance is indicated with asterisks $*p < 0.05$ and $**p < 0.01$. The graphing shows median, 25- and 75 percentile boxes and Tukey whiskers. Statistics: Kruskal Wallis test and Dunn's multiple comparisons test.

Supplementary figure 3. Mucosal CTH522 IgG

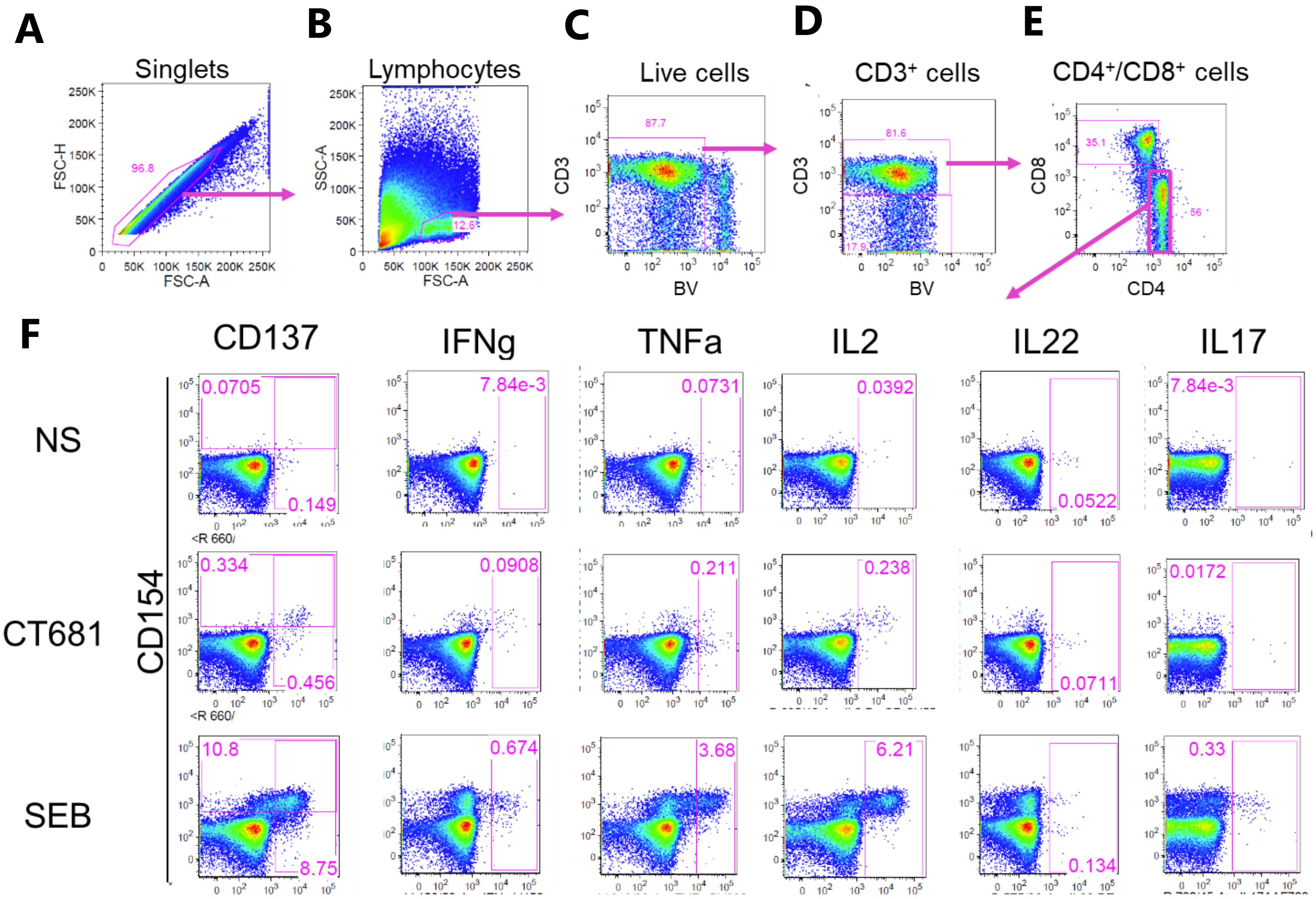

Supplementary figure 4. Gating strategy for Cytokine production analysis in CD4⁺ T cell by multiparameter flow cytometry. The FSC-A versu FSC-H gate isolate singlet cells (A), the FSC-A versus SSC-A gate distingued Lymphocytes from from macrophages and polymorphonuclear cells on the basis of morphology (B). CD4+ T cell were identified on lived cells (C) as $CD3^+CD4^+CD8^-$ cells (D-E). On the $CD4^+$ T cells, each $CD154^+$, $CD137^+$, $IFNg^+$, $TNFa^+$, $IL2^+$, $IL22^+$ and $IL17^+$ cells are gated for Boolean gate analysis and multifunctional analysis (F). The gating strategy is illustrated for one representative animal, PBMC are stimulated by CT681 peptides as an antigen specific stimulation, SEB as positive control or not stimulated as negative control.

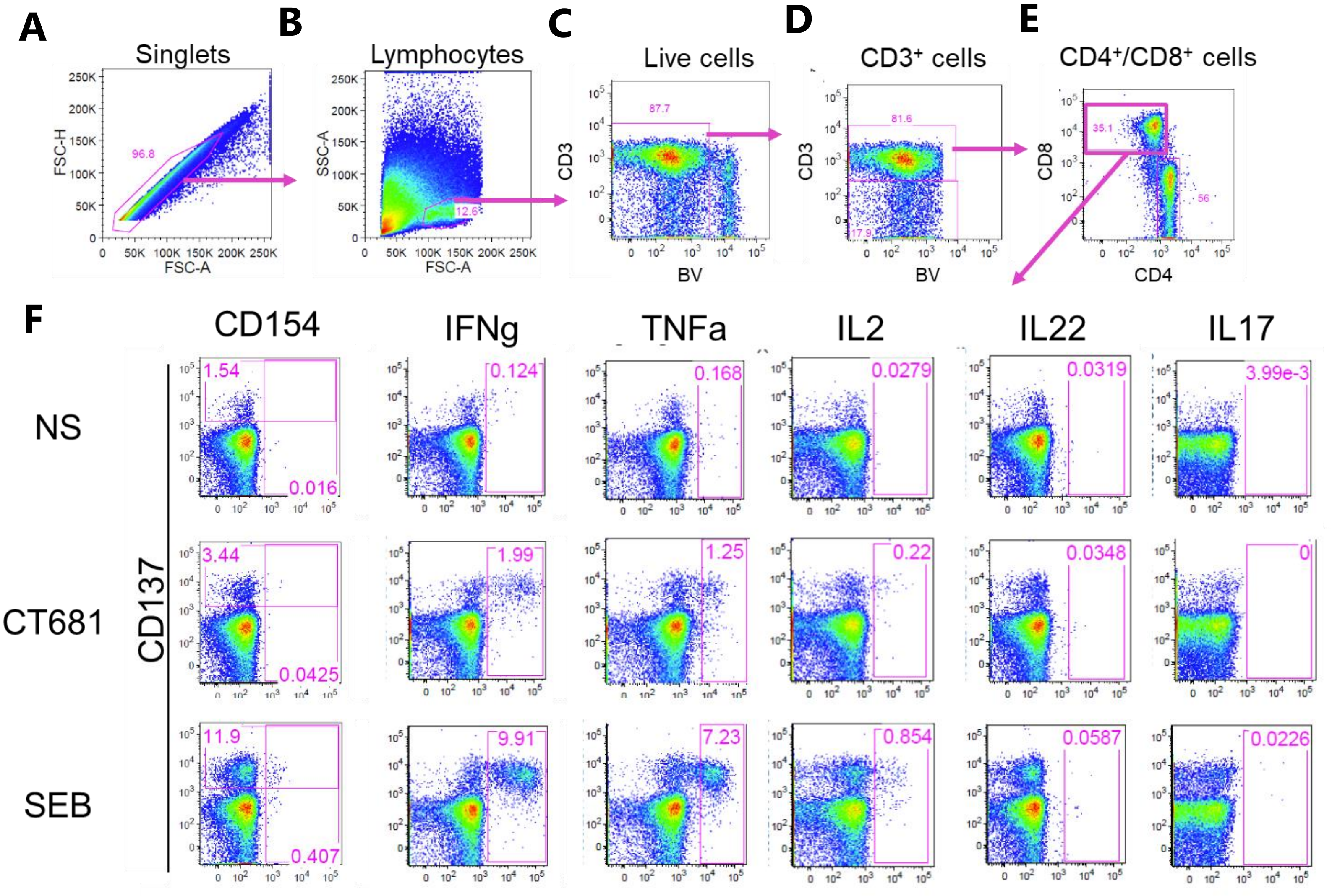

Supplementary figure 5. Gating strategy for Cytokine production analysis in CD8⁺ T cell by multiparameter flow cytometry. The FSC-A versu FSC-H gate isolate singlet cells (A), the FSC-A versus SSC-A gate distingued Lymphocytes from macrophages and polymorphonuclear cells on the basis of morphology (B). CD8+ T cell were identified on lived cells (C) as $CD3^+CD8^+CD4^-$ cells (D-E). On the $CD8^+$ T cells, each $CD154^+$, $CD137^+$, $IFNg^+$, $TNFa^+$, $IL2^+$, $IL22^+$ and $IL17^+$ cells are gated for Boolean gate analysis and multifunctional analysis (F). The gating strategy is illustrated for one representative animal, PBMC are stimulated by CT681 peptides as an antigen specific stimulation, SEB as positive control or not stimulated as negative control.

Supplementary Figure 6. Plasmid DNA Vaccine

Construction of the pcDNA3.1-MOMP

Vector: pcDNA3.1

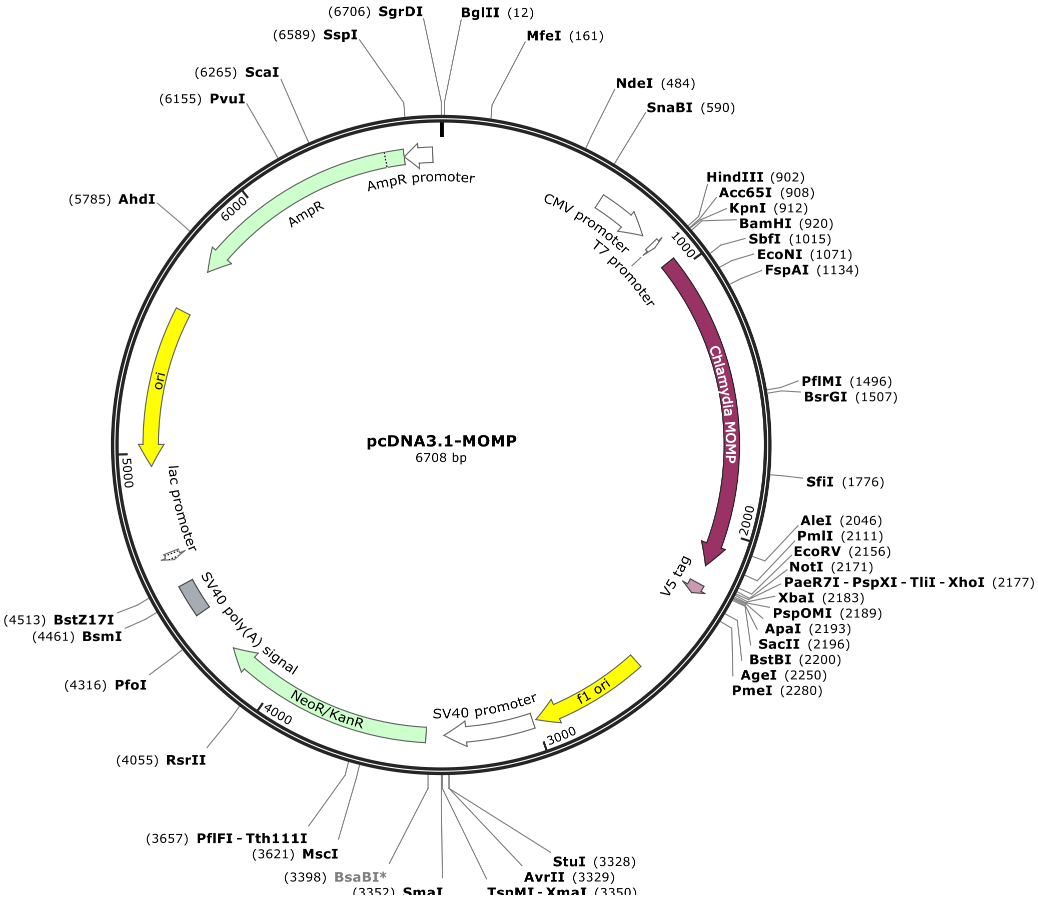

Supplementary figure 7. Vaccine antigen constructs
Recombinant Human Adenovirus serotype 5 - MOMP
rHuAd5-MOMP
Vector: pAL1112

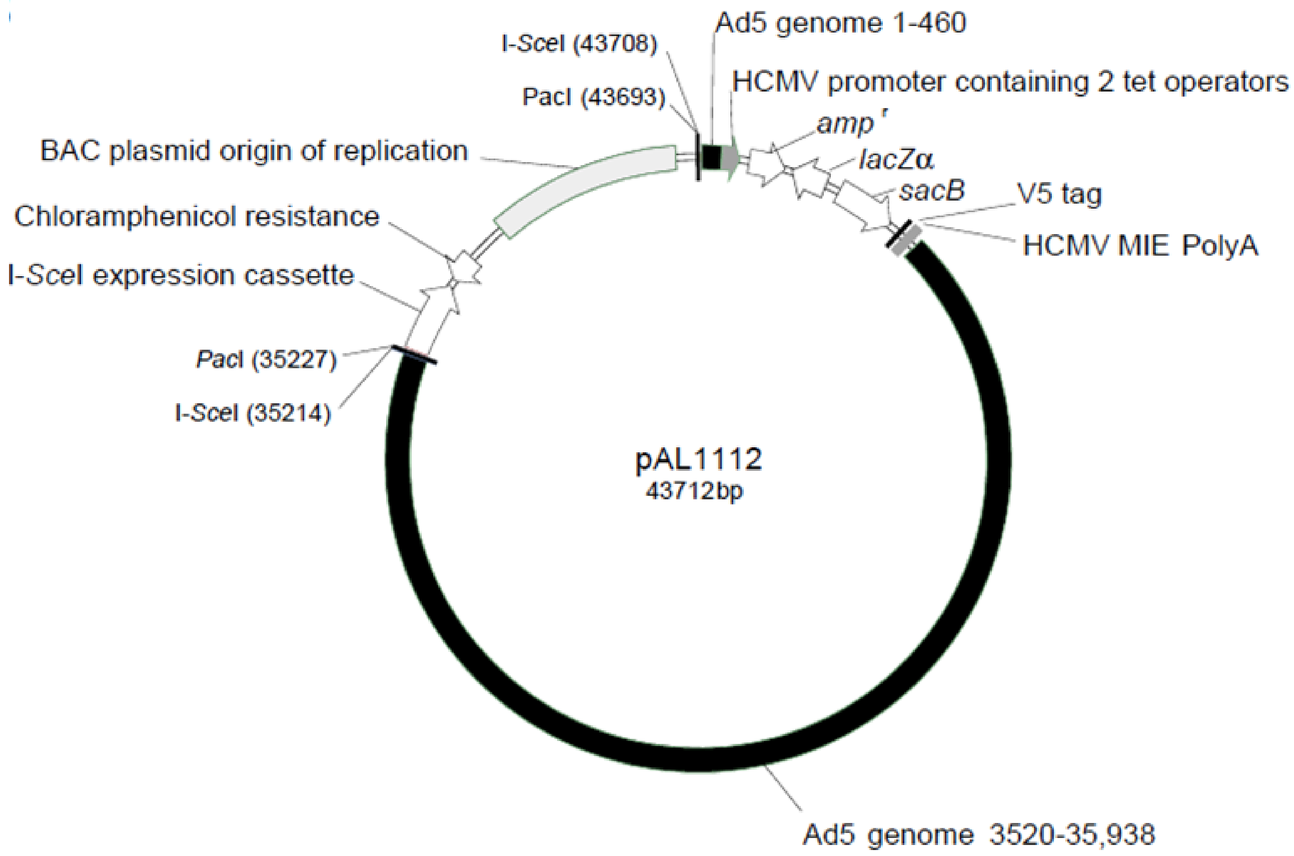

Supplementary figure 8. Vaccine antigen constructs
Modified Vaccinia Ankara (MVA)-MOMP
MVA-MOMP
Vector: vaccinia virus

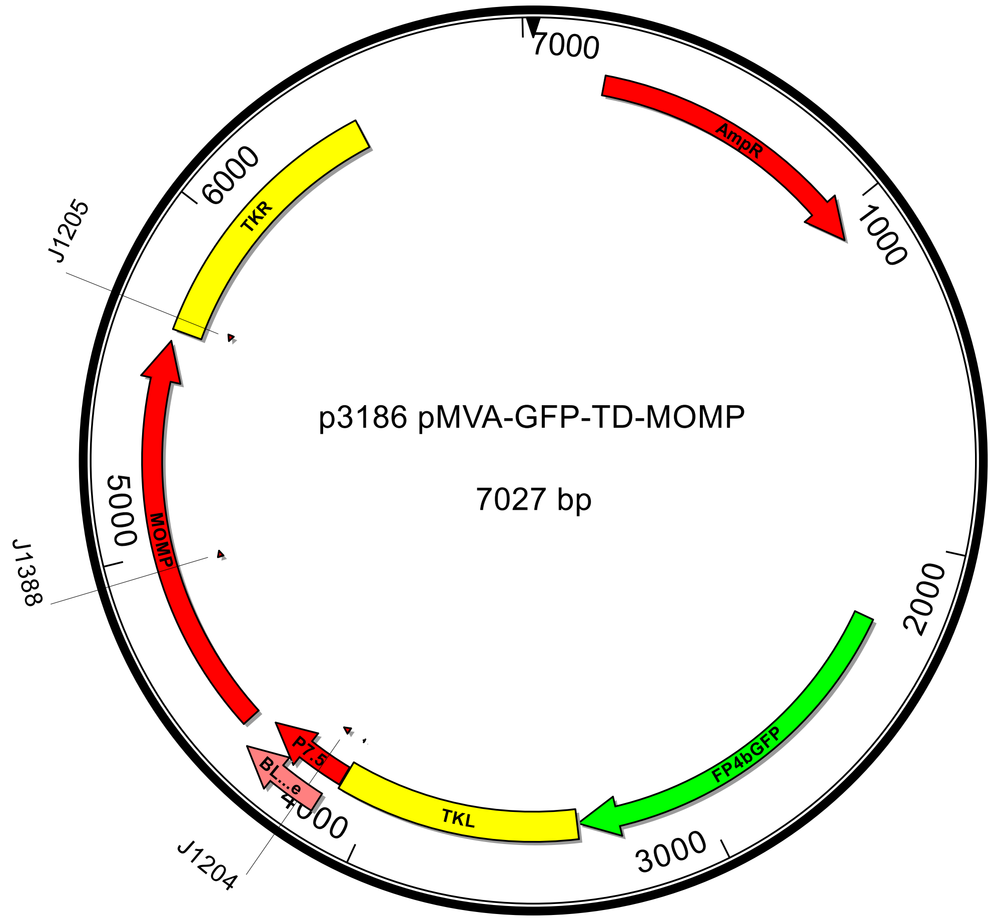

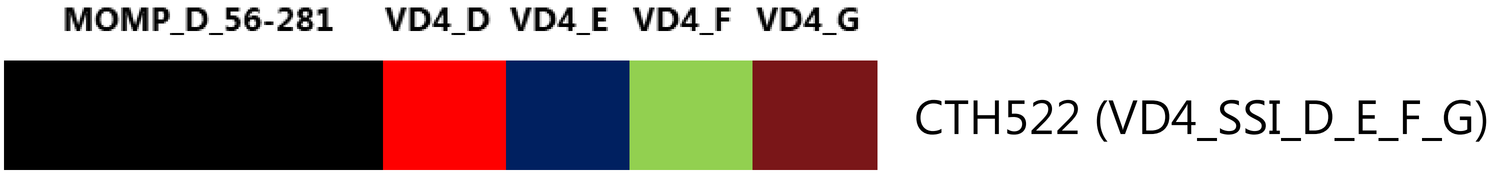

MHHHHHHDAISMRVGYYGDFVFDRVLKTDVNKEFQMGAKPTTDTGNSAAPST
LTARENPAYGRHMQDAEMFTNAASMALNIWDRFDVFSTLGATSGYLKGNSAS
FNLVGLFGDNENQKTVKAESVPNMSFDQSVVELYTDTTFAWSVGARAALWES
GSATLGASFQYAQSKPKVEELNVLSNAAEFTINKPKGYVGKEFPLDLTAGTD
AATGTKDASIDYHEWQASLALSYRLNMFTPYIGVKWSRASFDADTIRIAQPK
SATAIFDTTTLNPTIAGAGDVKTGAEGQLGDTMQIVSLQLNNMFTPYIGVKW
SRASFDADTIRIAQPKSATAIFDTTTLNPTIAGAGDVKASAEGQLGDTMQIV
SLQLNNMFTPYIGVKWSRASFDSDTIRIAQPRLVTPVVDITTLNPTIAGSGS
VAGANTEGQISDTMQIVSLQLNNMFTPYIGVKWSRASFDSNTIRIAQPKLAK
PVVDITTLNPTIAGSGSVVAANSEGQISDTMQIVSLQLN

Supplementary figure 9. The CTH522 vaccine construct

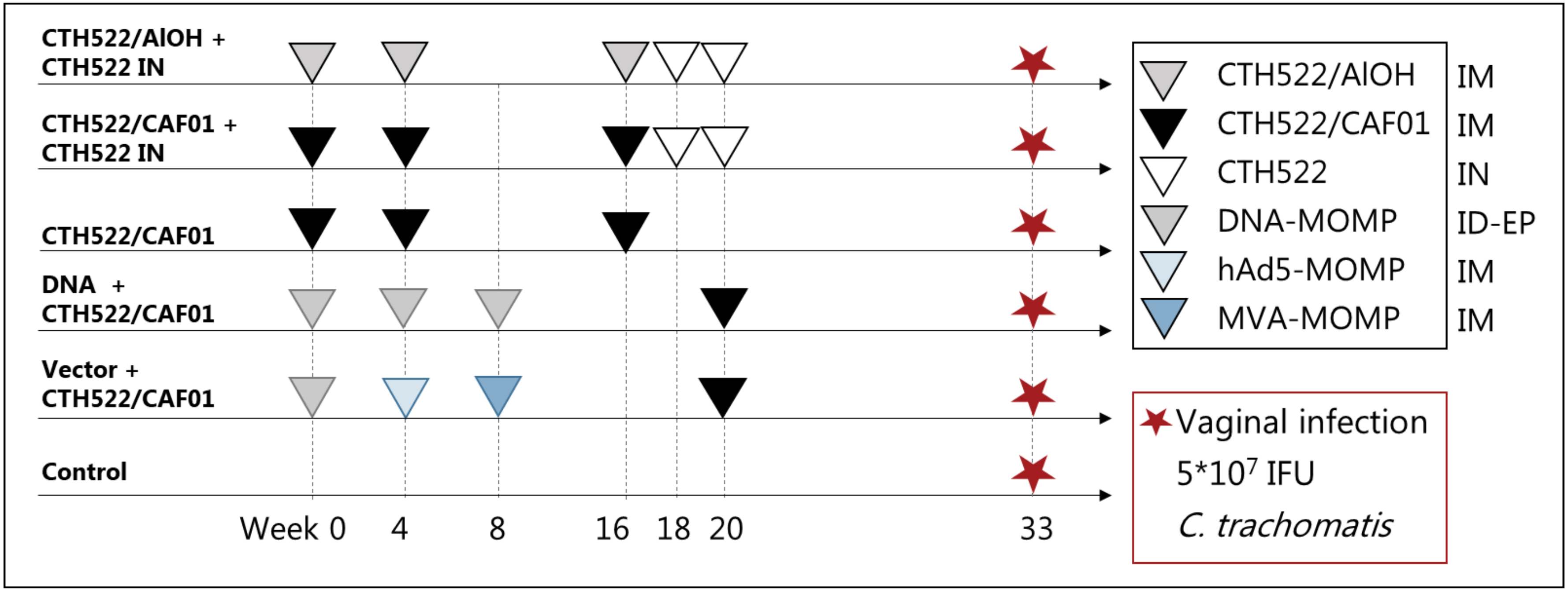

Supplementary figure 10. Overview of the experimental groups
